# The Stenopodainae (Hemiptera, Heteroptera) of Argentina

**DOI:** 10.3897/zookeys.452.6519

**Published:** 2014-11-05

**Authors:** Fernando Diez, María del Carmen Coscarón

**Affiliations:** 1 Universidad Nacional de La Pampa, Facultad de Ciencias Exactas y Naturales. Avenida Uruguay 151, (L 6300 CLB) Santa Rosa, La Pampa, Argentina; 2 División Entomología, Facultad de Ciencias Naturales y Museo, Universidad Nacional de La Plata, Paseo del Bosque, CP 1900, La Plata, Argentina

**Keywords:** Reduviidae, Stenopodainae, key, distribution, new record, Argentina

## Abstract

In Argentina, 10 genera and 33 species of Stenopodainae (Hemiptera: Reduviidae) have been recorded. Diagnoses of the genera, subgenera and species are given, and an illustrated key to genera is provided. Six species are new records for Argentina and an additional seven species represent new records for provinces.

## Introduction

The Stenopodainae are characterized by the presence of a large cell, usually pentagonal or hexagonal, in the venation of the hemelytra, formed by the cubital and postcubital veins and the apical and posterior cubital and postcubital crossveins (Barber 1930; Weirauch and Munro 2009). The antenniferous tubercles and juga (mandibular figs) are usually strongly produced anteriorly. The elongate and incrassate scapus is also an important subfamily character (Barber 1930; Schuh and Slater 1995).

This subfamily contains 113 genera with 713 species worldwide (Maldonado Capriles 1990). A total of 10 genera with 27 species have been recorded in Argentina (Coscarón in press). The Stenopodainae subfamily is monophyletic (Weirauch 2008, Weirauch and Munro 2009, Hwang and Weirauch 2012). This subfamily is phylogenetically closely related to the subfamily Triatominae and the genera *Zelurus* Burmeister and *Opisthacidius* Berg of the subfamily Reduviinae (Hwang and Weirauch 2012). Eggs are laid singly and loosely inside soil exposing their apices (Ambrose 1999); some species are nocturnal and can be captured by light traps (Villiers 1948 and personal observation).

Argentina – the geographical area considered in this report – lies in the Neotropical faunal region. The country covers an area of 2,791,810 km^2^ and is bordered by Uruguay, Brazil, Paraguay, Bolivia, and Chile. Approximately 75% of the country is occupied by arid and semiarid areas, but some places, such as the Yungas and Paranaense regions, are covered by rainforest.

The objective of this report is to provide an illustrated key of the genera of Stenopodainae from Argentina, including new diagnoses, geographical distribution records, and lists of species for each genus.

## Material and methods

This study is based on material provided by the Museo Argentino de Ciencias Naturales (MACN) and the Museo de La Plata (MLP) (http://heteroptera.myspecies.info), Argentina. We have followed the terminology of Barber (1930) and Giacchi (1970, 1974). Distance from the anterior margin of the eyes to the apex of the antenniferous tubercles is the anteocular region. Distance from the posterior margin of the eyes to the pronotal collar is the postocular region.

Images were taken with a digital camera (PANASONIC DMC-S3) and a Wild M-stereomicroscope. The material was compared with photographs of type from the Naturhistoriska Riksmuseet of Stockholm, Sweden (http://www.nrm.se) and the American Museum of Natural History of New York (http://www.amnh.org). The distributions we list for Argentina were taken from Coscarón (in press). We used the program DIVA-GIS 7.1.7 (http://www.diva-gis.org) and the distribution of those specimens for which global positioning system data were available to construct the maps.

## Results

Key to the genera of Stenopodainae for Argentina modified from Wygodzinsky and Giacchi (1994)

**Table:** 

1a	Scapus produced beyond insertion of the basiflagellomere (Fig. 1)	***Pnirontis* Stål** (Figs 28–31)
1b	Scapus not produced beyond insertion of basiflagellomere	2
2a	First labial segment approximately twice as long as the second and third segments combined (Fig. 2)	***Pygolampis* Germar** (Fig. 32)
2b	First labial segment equal to or shorter than second and third segments combined (Fig. 3)	**3**
3a	Prosternum behind coxae as long as or longer than coxae (Fig. 3)	**4**
3b	Prosternum behind coxae shorter than coxae, or coxae inserted at hind margin of prosternum (Fig. 4)	**7**
4a	Disc of anterior lobe of pronotum with 1+1 distinct tubercles (Figs 3, 5). First labial segment about as long as second segment (Fig. 3). Anterolateral angles of collar angles acutely spinous (Fig. 5). Fore coxae elongate cylindrical, about twice as long as wide (Fig. 3), hemelytral apical cubital and postcubital cross-vein obsolete (Fig. 26)	***Ocrioessa* Bergroth** (Fig. 26)
4b	Disc of fore lobe of pronotum without 1+1 distinct spine-like tubercles	**5**
5a	Apex of scutellum angularly raised or vertical (Fig. 6). Anteocular region as long as or slightly shorter than postocular region (Fig. 33). Two lines of spines on ventral side of head slightly surpassing the anterior and posterior margins of eyes, ventral spine about half or as long as posteroventral spines behind eyes (Fig. 7)	***Seridentus* Osborn** (Fig. 33)
5b	Apex of scutellum horizontal (Fig. 8). Anteocular region twice as long as postocular region. Spine on ventral side of head much smaller than the ventrolateral one behind eyes (Fig. 7)	***Ctenotrachelus* Stål** (Figs 17–19)
6a	Body and appendages with dense, adpressed pubescence and numerous tiny, erect bristles (Figs 4, 9, 34–36). Margins of head nearly parallel-sided, in dorsal view, abruptly constricted at neck (Fig. 9). Foretibiae with elongate fossula spongiosa	***Stenopoda* Laporte** (Figs 34–36)
6b	Body glabrous or variously pubescent but not as above	**7**
7a	Anteocular and postocular regions of equal length (Fig. 10). Body elongate fusiform, often five times or over five times as long as maximum width (Figs 22, 23). Male genitalia with cuplike posterior extension of pygophore completely covering parameres. Female genital area narrowly pointed posteriorly	***Gnathobleda* Stål** (Figs 22, 23)
7b	Anteocular region longer than postocular region (Fig. 11). Body not elongate fusiform, broader, always less than five times as long as maximum width (Fig. 27). Male genitalia with parameres not covered, clearly visible. Female genital area not narrowly pointed posteriorly	**8**
8a	Forefemora strongly incrassate, at least twice as thick as middle and hind femora (Fig. 27). Foretibia without distinct fossula spongiosa, or the latter not longer than diameter of tibia (Fig. 12)	***Oncocephalus* Klug** (Fig. 27)
8b	Forefemora slender, less than twice as thick as mid and hind femora (Fig. 20)	**9**
9a	Jugum subcylindrical, parallel, round apically, projecting well beyond apex of head (Fig. 13). Tibiae of hind legs with setae of a length less than twice the diameter of the tibia (Fig. 14)	***Diaditus* Stål** (Figs 20, 21)
9b	Jugum triangular bifurcated, apically sharp, not projecting beyond apex of head (Fig. 15). Tibae of hind legs with setae of length equal to four or five times the diameter of the tibia (Fig. 16)	***Narvesus* Stål** (Figs 24, 25).

**Figures 1–8.**
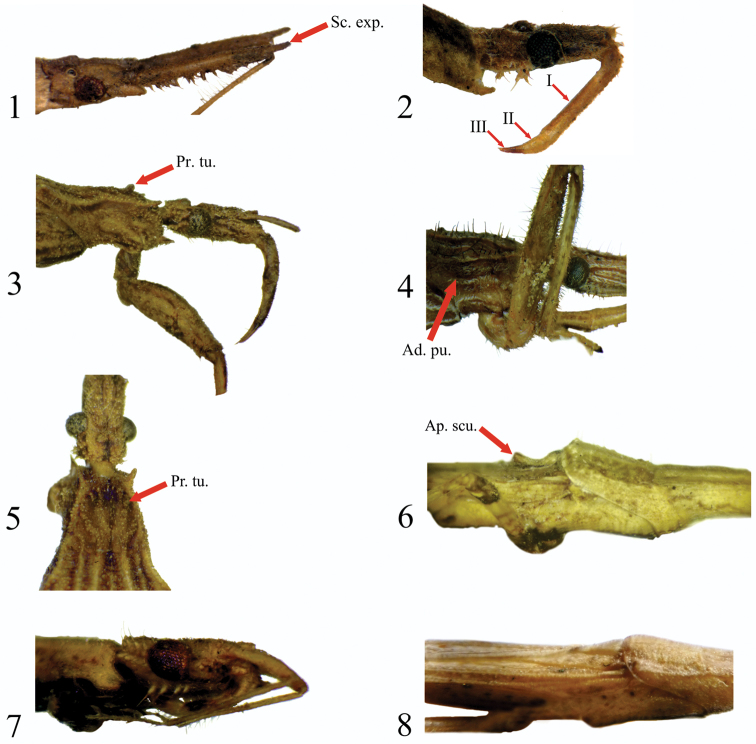
Generic characters. **1** Head *Pnirontis stali* **2** Head *Pygolampis spurca* **3** Pronotum lateral view *Ocrioessa cornutulus* **4** Pronotum lateral view *Stenopoda guaranitica* **5** Pronotum dorsal view *Ocrioessa cornutulus* **6** Scutellum lateral view *Seridentus maculosus* **7** Head and pronotum lateral view *Seridentus maculosus* **8** Pronotum lateral view *Ctenotrachelus* sp. (Ad pu: adpressed pubescence; Ap scu: apex of scutellum; Pr tu: pronotal tubercles; Sc exp: expansions of scapus; I: first labial segment; II: second labial segment; III: third labial segment).

**Figures 9–16.**
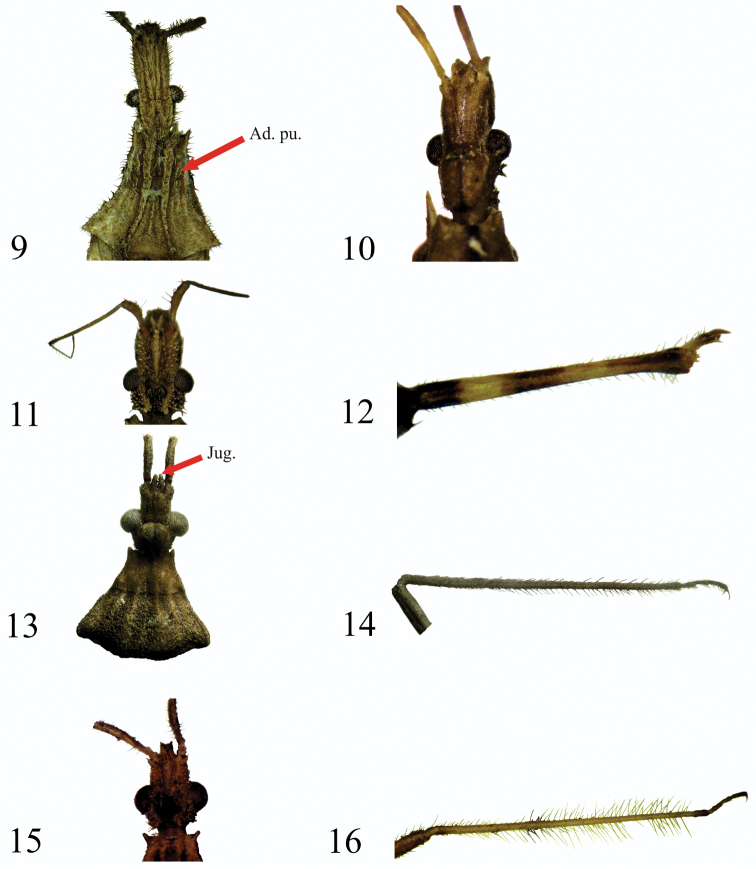
Generic characters. **9** Head dorsal view *Stenopoda guaranitica* **10** Head dorsal view *Gnathobleda toba* **11** Head dorsal view *Oncocephalus validispinis* **12** Tibiae ventral view *Oncocephalus validispinis* **13** Head and pronotum dorsal view *Diaditus latulus* **14** Tibiae dorsal view *Diaditus latulus* **15** Head dorsal view *Narvesus carolinensis* **16** Tibiae dorsal view *Narvesus carolinensis*. (Ad pu: adpressed pubescence; Jug: juga).

**Figures 17–36.**
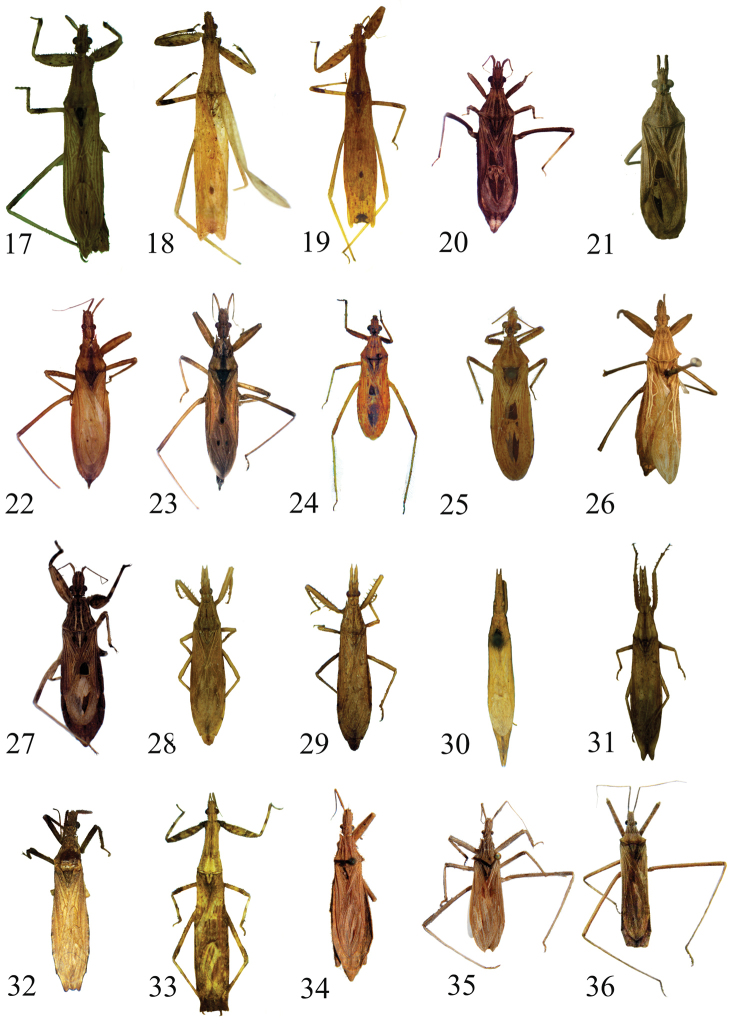
Dorsal view. **17** *Ctenotrachelus minor* Barber **18** *Ctenotrachelus striatus* Barber **19** *Ctenotrachelus testaceus* Barber **20** *Diaditus pilosicornis* Bergroth **21** *Diaditus latulus* Barber. **22** *Gnathobleda toba* Giacchi **23** *Gnathobleda litigiosa* Stål **24** *Narvesus carolinensis* Stål **25** *Narvesus minor* Barber **26** *Ocrioessa cornutulus* (Berg) **27** *Oncocephalus validispinis* Reuter **28** *Pnirontis edentula* (Berg) **29** *Pnirontis infirma* Stål **30** *Pnirontis scorpiona* (Berg) **31** *Pnirontis stali* (Mayr) **32** *Pygolampis spurca* Stål **33** *Seridentus maculosus* (Haviland) **34** *Stenopoda cana* Stål **35** *Stenopoda guaranitica* Giacchi **36** *Stenopoda subinermis* Stål.

**Figures 37–41.**
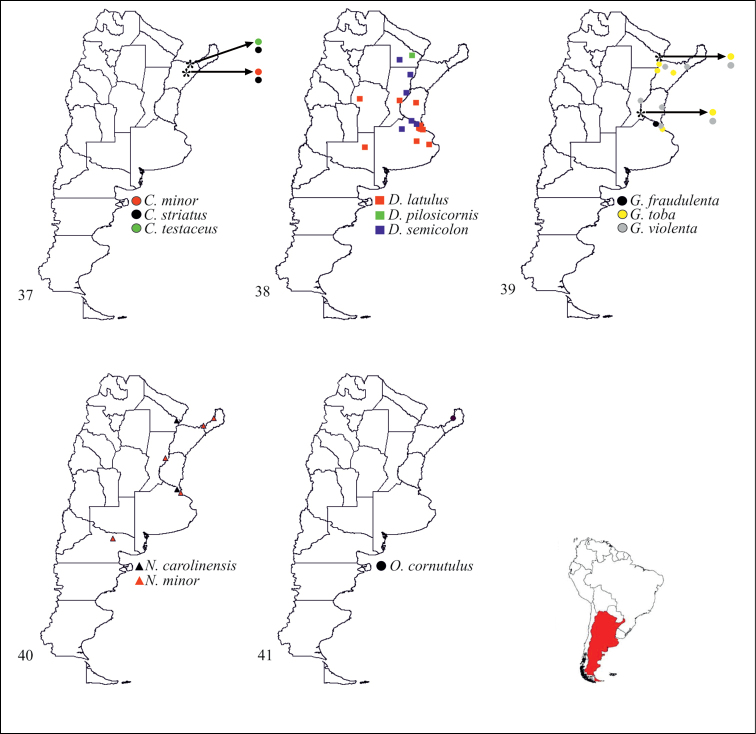
Geographical distributions of species of Stenopodainae in Argentina: **37** *Ctenotrachelus* Stål **38** *Diaditus* Stål **39** *Gnathobleda* Stål **40** *Narvesus* Stål **41** *Ocrioessa* Bergroth.

**Figures 42–46.**
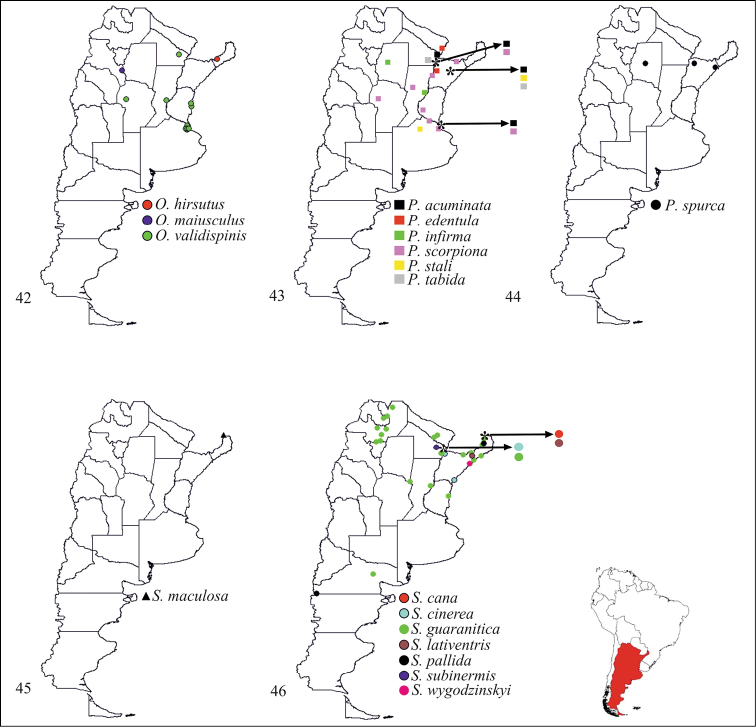
Geographical distributions of species of Stenopodainae in Argentina: **42** *Oncocephalus* Klug **43** *Pnirontis* Stål **44** *Pygolampis* Germar **45** *Seridentus* Osborn **46** *Stenopoda* Laporte.

## Taxonomy

### Ctenotrachelus

Taxon classificationAnimaliaHemipteraReduviidae

Stål

Ctenotrachelus Stål, 1868: 127.

#### Type species.

*Ctenotrachelus macilentus* Stål, 1872, subsequent monotypy.

#### Diagnosis.

(After Barber 1930, Giacchi 1985, Maldonado Capriles 1994a) Anteocular region twice as long as postocular region. Setigerous tubercle on ventral side of head much smaller than the ventrolateral tubercle behind eyes. Pronotum longer than wide, with the anterior lobe much longer than posterior one. Scutellar spine horizontal, metascutellar spine small. Fore femora sligthly incrassate. Anterior legs with third tarsal segment longer than first and second together.

### Ctenotrachelus minor

Taxon classificationAnimaliaHemipteraReduviidae

Barber

Ctenotrachelus minor Barber, 1930: 188, 200.

#### Diagnosis.

(After Barber 1930, Maldonado Capriles 1995) Scapus three times as long as anteocular margin. Pronotum less than twice as long as head. Prefemur strongly incrassate.

#### Material examined.

Corrientes: 1♂ (MLP) Colonia Carlos Pellegrini (28°31'54.0984"S, 57°9'49.8204"W), Coscarón M. coll.

#### Observation.

New record for Argentina.

### Ctenotrachelus striatus

Taxon classificationAnimaliaHemipteraReduviidae

Barber

Ctenotrachelus striatus Barber, 1930: 197; Giacchi 1985: 67; Coscarón 2003: 361; Melo et al. 2004: 61.

#### Diagnosis.

(After Barber 1930) Preocular region of head one third longer than postocular one. Lateral margins of pronotum unarmed. First two ventral abdominal segments carinate.

#### Material examined.

Corrientes: 1♂ (MLP) Colonia Carlos Pellegrini, Coscarón M. coll.

#### Distribution in Argentina.

Corrientes: Colonia Carlos Pellegrini (28°31'54.0984"S, 57°9'49.8204"W), Ituzaingó (27°40'30.8742"S, 56°48'13.9428"W).

### Ctenotrachelus testaceus

Taxon classificationAnimaliaHemipteraReduviidae

Barber

Ctenotrachelus testaceus Barber, 1930: 189.

#### Diagnosis.

(After Barber 1930) Postocular and preocular regions of head nearly equal or postocular region shorter than preocular one. Head behind eyes armed with three simple spines. Lateral margins of pronotum unarmed. First four segments of ventral abdominal segments carinate.

#### Material examined.

Corrientes: 1♂ (MLP) Ituzaingó (27°40'30.8742"S, 56°48'13.9428"W), Coscarón M. col.

#### Observation.

New record for Argentina.

### Diaditus

Taxon classificationAnimaliaHemipteraReduviidae

Stål

Diaditus Stål, 1859: 383.

#### Type species.

*Diaditus semicolon* Stål 1859.

#### Diagnosis.

(After Barber 1930, Giacchi 1973) Preocular region longer than postocular region. Juga long, robust and blunt apex, well extended beyond apices of antenniferous tubercles. Scapus shorter than head. First labial segment nearly equal to the second and third segments together. Hind tibiae with short setae, never reaching twice the diameter of the tibia. Anterior femora scarcely incrassate. Abdomen in ventral view with a median longitudinal carina, extending from sternum II to VI.

### Diaditus latulus

Taxon classificationAnimaliaHemipteraReduviidae

Barber

Diaditus latulus Barber, 1930: 221; Wygodzinsky 1949: 66; Dispons 1971: 274; Giacchi 1982: 26; Maldonado Capriles 1990: 501; Martin-Park and Coscarón 2011: 56.

#### Diagnosis.

(After Barber 1930, Giacchi 1982) Head short, less than twice as long as wide. Head shorter than pronotum. Males with setae in the ventral and lateral internal face of Pedicellus, seta length equal to twice the diameter of Pedicellus. Juga short, robust, subparallel, not reaching 1/4 of scapus in males, but reaching almost half in females. Collar angles blunt.

#### Material examined.

La Pampa: 1♂ (MLP) Santa Rosa (36°36'56.8902"S, 64°17'49.7106"W), Diez F. Col.; Córdoba: 3♂ (MACN) Departamento Calamuchita: El Sauce (31°6'0.3312"S, 64°19'0.0084"W).

#### Distribution in Argentina.

Buenos Aires: Daguerre (34°39'17.4636"S, 58°28'53.2878"W), Delta (34°14'12.4188"S, 58°34'10.1598"W), Dolores (36°18'53.2044"S, 57°40'47.7798"W), Hurlingham (34°35'52.4004"S 58°38'8.7"W), Baradero (33°48'30.4704"S 59°30'19.6986"W), Rosas (35°57'56.7714"S, 58°56'24.1944"W), San Miguel (34°32'39.4152"S, 58°42'59.457"W), Wilde (34°42'15.7752"S, 58°19'13.623"W); Córdoba: Sierras (31°26'20.4678"S, 64°50'4.0992"W); Entre Ríos: Villaguay (31°51'53.0244"S, 59°2'8.5956"W); Mendoza; Salta; San Juan; Santa Fe: Bridarolli (31°37'56.5998"S, 60°41'58.0518"W).

#### Observation.

First record for La Pampa province.

### Diaditus pilosicornis

Taxon classificationAnimaliaHemipteraReduviidae

Bergroth

Diaditus pilosicornis Bergroth, 1907: 50; Melo et al. 2011.

#### Diagnosis.

(After Barber 1930, Giacchi 1982) Males with setae on ventral and lateral internal face of Pedicellus, seta length three times the diameter of Pedicellus. Juga reaching more than 1/3 of scapus in males and more than half in females. Prosternum glabrous, if tubercles or setae are present, these are scarce and conspiscuous. Collar angle obtuse. Fore femora in the ventral surface, basally with one spiniferous tubercle, the height is twice or more than setigerous tubercles of the trochanter.

#### Material examined.

Chaco: 1♀ (MLP) Chaco National Park.

#### Distribution in Argentina.

Chaco: Chaco National Park (26°48'24.9984"S, 59°26'36.4986"W).

### Diaditus semicolon

Taxon classificationAnimaliaHemipteraReduviidae

Stål

http://heteroptera.myspecies.info/taxonomy/term/1828

http://www2.nrm.se/en/het_nrm/s/diaditus_semicolon.html

Diaditus semicolon Stål, 1859: 383; Berg 1879: 278; Lethierry and Severin 1896: 86; Pennington 1921: 22; Barber 1930: 220; Giacchi 1982: 22; Maldonado Capriles 1990: 501; Nanni et al. 2011: 34; Dellapé and Carpintero 2012: 130.
Diaditus annulipes Berg, 1883: 112; Lethierry and Severin 1896: 86; Pennington 1921: 22; Coscarón 1998: 2.

#### Diagnosis.

(After Barber 1930, Giacchi 1982, Blinn 2009) Males with setae on ventral and lateral internal face of pedicellus, seta length three times the diameter of pedicellus. Juga reaching 1/5 of scapus in males and 1/3 in females. Fore femora with one or two setigerous tubercles, not larger than setigerous tubercles of the trochanter.

#### Material examined.

1♂ (MLP) Typus *Diaditus annulipes* Berg synonymized by Wygodzinsky 1949, 1:66, 67. (Geographic origin not given). Salta: 3♀, Embarcación (23°12'42.0798"S, 64°6'4.9026"W), 2♂ (MLP) City (24°47'6"S, 65°24'32.9904"W). Mendoza ♀ (MLP) Typus *Diaditus annulipes* Berg synonymized by Wygodzinsky 1949, 1:66–67.

#### Distribution in Argentina.

Buenos Aires: Baradero (33°48'30.4704"S, 59°30'19.6986"W), Chacabuco (34°38'22.4304"S, 60°28'9.9726"W), Partido de Campana: Delta del Paraná (34°9'9.8166"S, 58°58'11.136"W); Córdoba; Catamarca; Chaco: San Bernardo (27°17'18.6072"S, 60°42'45.6516"W), Tandil; Corrientes; Entre Ríos; Formosa; Jujuy; La Pampa; La Rioja; Mendoza; Misiones; Neuquén; Salta; San Juan; San Luis; Santa Fe: Colonia Mascías (30°48'7.6032"S, 60°0'19.6266"W), Departamento General Obligado, Lanteri (28°50'27.765"S, 59°38'9.981"W); Santiago del Estero; Tucumán.

#### Observation.

First record for Salta province.

#### Remarks.

The species currently assigned to the taxon is listed in Coscarón et al. (2014).

### Gnathobleda

Taxon classificationAnimaliaHemipteraReduviidae

Stål

Gnathobleda Stål, 1859: 380.

#### Type speccies.

*Gnathobleda fraudulenta* Stål 1859.

#### Diagnosis.

(After Wygodzinsky and Giacchi 1986) Sericeous pilosity. Anteocular and postocular portions of equal length. Large, pointed, laterally compressed juga. Presence of 1+1 conspicuous tubercles on the pronotum. More or less developed posterior projections on the connexival segments.

#### Note.

Wygodzinsky and Giacchi (1986) synonymised *Pnohirmus* Stål, with *Gnathobleda* Stål. Latter, Wygodzinsky and Giacchi (1994) in the key to the genera of the Stenopodainae of the new world they included the subgenera Ganthobleda (Gnathobleda) and Gnathobleda (Pnohirmus). In this article they did not mentioned the species for each subgenera. We do not use the subgenera of *Gnathobleda* due to this confusion.

### Gnathobleda fraudulenta

Taxon classificationAnimaliaHemipteraReduviidae

Stål

Gnathobleda fraudulenta Stål, 1859; Nanni et al. 2011: 34.

#### Diagnosis.

(After Wygodzinsky and Giacchi 1986) Head with a simple setigerous tubercle. Juga triangular. Prosternal processes conspicuous, spinelike. Undersurface of fore femora with two rows of processes.

#### Distribution in Argentina.

Buenos Aires: Partido de Campana: Delta del Paraná (34°9'9.8166"S, 58°58'11.136"W).

### Gnathobleda litigiosa

Taxon classificationAnimaliaHemipteraReduviidae

Stål

http://www2.nrm.se/en/het_nrm/l/gnathobleda_litigiosa.html

Gnathobleda litigiosa Stål, 1862: 442.

#### Diagnosis.

(After Wygodzinsky and Giacchi 1986) Length less than 14 mm. Genae conspicuously projecting beyond base of rostrum. Connexival segments light-colored with apical portion dark. Undersurface of fore femora with two series of processes, one setigerous, one spiniferous.

#### Material examined.

2♂ (MLP) between Corrientes and Formosa (unspecified locality).

#### Observation.

New record for Argentina.

### Gnathobleda toba

Taxon classificationAnimaliaHemipteraReduviidae

Giacchi

Gnathobleda toba Giacchi, 1970: 126; Maldonado Capriles 1990: 503; Melo et al. 2004: 61.

#### Diagnosis.

(After Wygodzinsky and Giacchi 1986) Total length 14 mm or more. Some of the sublateral setigerous spines of the postocular region of the head bifurcate. Genae conspicuously projecting beyond base of rostrum, connexival segments concolorous. Undersurface of fore femora with one series of spiniferous processes.

#### Material examined.

Buenos Aires: 1♂ (MLP) La Plata. Corrientes: 2♂ 2♀ (MLP) Bella Vista (28°30'27.8274"S, 59°2'39.6492"W), 1♂ (MLP) between Corrientes and Formosa (unspecified locality). Santa Fe: 1♂ (MLP) Rosario.

#### Distribution in Argentina.

Buenos Aires: Buenos Aires City (34°36'13.5102"S, 58°22'53.4678"W), La Plata (34°55'8.9616"S, 57°57'21.495"W); Chaco: General Vedia (26°55'58.728"S, 58°39'41.3958"W), Río de Oro (26°56'6.0858"S, 58°40'19.5414"W); Corrientes: Bella Vista, Colonia Carlos Pellegrini (28°32'5.4312"S, 57°10'27.5196"W).

#### Observation.

First record for Santa Fe.

### Gnathobleda violenta

Taxon classificationAnimaliaHemipteraReduviidae

(Stål)

Pnohirmus violentus Stål, 1859: 384; Giacchi 1985: 66; Coscarón 2003: 361.
Gnathobleda violenta Wygodzinsky and Giacchi, 1986: 141.

#### Diagnosis.

(After Wygodzinsky and Giacchi 1986) Sublateral setigerous spines of postocular region of the head absent, simple or at most fused at base. Genae not conspicuously projecting beyond base of rostrum. Head without setigerous spines. Juga imperceptible in lateral view. Prosternal processes small, rounded, underside of femora with one row of spiniferous processes.

#### Distribution in Argentina.

Buenos Aires: Delta (34°14'12.4188"S, 58°34'10.1598"W); Chaco: General Vedia (26°55'59.1234"S, 58°39'42.015"W), Río de Oro (26°56'6.0792"S, 58°40'19.5564"W); Corrientes: Manantiales (27°55'17.2878"S, 58°6'0.2874"W), Apóstol; Entre Ríos: Primero de Mayo (32°15'24.21"S, 58°25'22.5588"W); Santa Fe: Bridarolli, Piquete (31°34'19.6932"S, 60°43'19.023"W), Rosario (32°57'30.276"S, 60°39'32.688"W).

### Narvesus

Taxon classificationAnimaliaHemipteraReduviidae

Stål

Narvesus Stål, 1859: 384.

#### Type species.

*Narvesus carolinensis* Stål, 1859.

#### Diagnosis.

(After Barber 1930, Giacchi 1973, Giacchi 1974) Juga acute at the tip and divergent, never extending beyond the length of tylus. Scapus shorter than the head. Hind legs with very long setae on the tibia, four or five times the diameter of the tibia.

### Narvesus carolinensis

Taxon classificationAnimaliaHemipteraReduviidae

Stål

Narvesus carolinensis Stål, 1859: 385; Diez and Coscarón 2014: 290.

#### Diagnosis.

(After Barber 1930, Giacchi 1974) Anterior and middle tibia bifasciate. Fore femora without a row of spiniform tubercles on ventral face.

#### Material examined.

Buenos Aires: 1♀ (MLP) Olivos; Chaco: 1♂ (MACN) Río Oro.

#### Distribution in Argentina.

Buenos Aires: Olivos (34°30'39.1356"S, 58°29'44.7354"W); Chaco: Río Oro (26°56'6.0792"S, 58°40'19.5564"W).

### Narvesus minor

Taxon classificationAnimaliaHemipteraReduviidae

(Barber)

Narvesus minor Barber, 1930: 224; Giacchi 1974: 62; Maldonado Capriles 1990: 508; Carpintero 2009: 299; Diez and Coscarón 2014: 294.

#### Diagnosis.

(After Barber 1930, Giacchi 1974) Anterior and mid tibiae trifasciate. Fore femora with a row of spiniform tubercles on ventral face.

#### Material examined.

Santa Fe: 1♂ (MACN) Colonia Mascías; Neuquén: 1♂ (MLP) (unspecified locality).

#### Distribution in Argentina.

Buenos Aires: Parque Costero del Sur (35°16'22.6266"S, 57°15'50.724"W); Misiones: Bocceti, Montecarlo (26°34'30.0648"S, 54°45'33.4542"W), Zaimán (27°25'6.801"S, 55°53'40.47"W); Neuquén; Río Negro: Lamarque (39°25'12.2982"S, 65°42'0.9324"W). Santa Fe: Colonia Mascías (30°48'1.9362"S, 60°0'48.6138"W).

### Ocrioessa

Taxon classificationAnimaliaHemipteraReduviidae

Bergroth

Ocrioessa Bergroth, 1918: 312.

#### Type species.

Reduvius (Oncocephalus) notatus Klug 1830.

#### Diagnosis.

(After Barber 1930, Giacchi 1985) First labial segment about as long as second segment. Posterior ocular region shorter than anteocular region. Pronotum longer than wide, with deep transverse groove before the half. Disc of fore lobe of pronotum with 1 +1 distinct tubercles. Apical angles of segments II to VI terminated in triangular lobes, apical angles of segment VII ending in two acute lobes directed posteriorly.

### Ocrioessa cornutulus

Taxon classificationAnimaliaHemipteraReduviidae

(Berg)

http://heteroptera.myspecies.info/taxonomy/term/2052

Rhyparoclopius cornutulus Berg, 1879: 277; Lethierry and Severin 1896: 85; Pennington 1921: 22; Coscarón 1998: 509.
Ocrioessa cornutulus Giacchi 1985: 68; Maldonado Capriles 1990: 509.

#### Diagnosis.

(After Barber 1930, Giacchi 1985) Diameter of the gula much wider, being about twice as wide as the diameter of base of second labial segment. Scapus about twice as long as the preocular region. Discal spines of anterior lobe situated before the constriction with tubercles reduced.

#### Material examined.

Misiones: 1♀ (MLP) Montecarlo.

#### Distribution in Argentina.

Chaco; Misiones: Montecarlo (26°34'23.4294"S, 54°45'29.7462"W).

#### Remarks.

The species currently assigned to the taxon is listed in Coscarón et al. (2014).

### Oncocephalus

Taxon classificationAnimaliaHemipteraReduviidae

Klug

Reduvius (Oncocephalus) Klug, 1830: 2. Type species: Reduvius (Oncocephalus) notatus Klug, 1830.
Oncocephalus Fieber, 1860: 42.

#### Diagnosis.

(After Giacchi, 1984) Body oval. Eyes of male large, eyes of female smaller, with several rather distinct setigerous tubercles behind eyes. Antennae and tibiae with long setae, particularly in males. Basal segment of rostrum shorter than the two apical segments together. Fore femora strongly incrassate and distinctly spinose (with one or two row(s) of teeth on the ventral side).

### Oncocephalus hirsutus

Taxon classificationAnimaliaHemipteraReduviidae

Giacchi

Oncocephalus hirsutus Giacchi, 1984: 57; Maldonado Capriles 1990: 514.

#### Diagnosis.

(After Giacchi 1984, Barber 1930) Pedicellus with long setae, more than three times the diameter of segment in males. The lateral tubercles of pronotum covered with stiff setae. Fore femora with seven spines ventrally and dorsally covered by conspicuous setigerous tubercles.

#### Distribution in Argentina.

Misiones: Loreto (27°18'59.925"S, 55°31'58.8462"W).

### Oncocephalus maiusculus

Taxon classificationAnimaliaHemipteraReduviidae

Giacchi

Oncocephalus maiusculus Giacchi, 1984: 58; Maldonado Capriles 1990: 515; Martin-Park and Coscarón 2011: 57.

#### Diagnosis.

(After Barber 1930, Giacchi 1984) Scapus, in the male, (in dorsal internal lateral view) with setae on the distal half or basal third. Setae length equal to half the diameter of scapus. Posterior lobe of pronotum brown and smooth.

#### Distribution in Argentina.

Catamarca: Los Alamitos (28°28'59.4372"S, 65°13'8.2698"W).

### Oncocephalus validispinis

Taxon classificationAnimaliaHemipteraReduviidae

Reuter

Oncocephalus validispinis Reuter, 1882: 714; Giacchi 1984: 55; Maldonado Capriles 1990: 521; Martin-Park and Coscarón 2011: 57; Melo et al. 2011.
Oncocephalus mazzai Costa Lima, 1941: 342; Wygodzinsky 1949: 67.

#### Diagnosis.

(After Barber 1930, Giacchi 1984) Scapus (in dorsal internal lateral view) with three setae shorter than the diameter of scapus in male and two in females. Setae shorter than the diameter of scapus. Posterior lobe of pronotum with medial longitudinal lines and carina. Two light brown bands on either side of carina.

#### Material examined.

Chaco: 1♂ (MLP) Chaco National Park.

#### Distribution in Argentina.

Buenos Aires: Delta (34°14'12.4188"S, 58°34'10.1598"W), Haedo (34°38'39.714"S, 58°35'43.6272"W), Hurlingham (34°35'52.4004"S, 58°38'8.7"W), Morón (34°39'21.0996"S, 58°37'0.195"W), San Miguel (34°32'34.9614"S, 58°42'43.0812"W), Villa Ballester (34°32'57.231"S, 58°33'31.6902"W), Ciudad Universitaria (34°34'46.5018"S, 58°24'17.2218"W); Chaco: Chaco National Park (26°48'24.9984"S, 59°26'36.4986"W); Córdoba: Sierras (31°26'20.4678"S, 64°50'4.0992"W); Entre Ríos: Colón (32°13'30"S, 58°8'40.1922"W), El Palmar (31°52'2.5932"S, 58°12'31.953"W); Santa Fe: Piquete (31°34'17.9826"S, 60°42'32.6736"W); Santiago del Estero.

### Pnirontis

Taxon classificationAnimaliaHemipteraReduviidae

Stål 1859

Pnirontis Stål, 1859: 381.

#### Type species.

*Pnirotis scutellaris* Stål 1859; subsequent designation by Van Duzee 1916.

#### Diagnosis.

(After Barber 1930, Giacchi 1985, Giacchi 1988a) Body elongate longitudinally, fusiform and depressed. First labial segment almost three times longer than the second and third together, the second almost twice as long as the third. Scapus strongly incrassate, extended in an apical process that extends beyond the insertion of the second segment.

### Pnirontis (Centromelus)

Taxon classificationAnimaliaHemipteraReduviidae

Stål, 1868

#### Diagnosis.

(After Wygodzinsky and Giacchi 1994) Antenniferous tubercles unarmed, or provided with minute spines. First segment of mid and hind tarsi shorter than second. Posterior angles of connexival segments varied.

#### Type species.

Pnirontis (Centromelus) spinosissimus Stål, 1859; subsequent designation by Van Duzee (1916).

### Pnirontis acuminata

Taxon classificationAnimaliaHemipteraReduviidae

Barber

Pnirontis acuminata Barber, 1930: 156; Giacchi 1985: 64; Giacchi 1988a: 6.
Pnirontis (Centromelus) acuminata Melo et al. 2004: 61.

#### Diagnosis.

(After Barber 1930, Giacchi 1985, Maldonado Capriles 1986, 1994b) Head longer than pronotum. Scapus equal to length of preocular margin of head. Spines of fore femora long, two or three times as long as diameter of femur. Connexivum marked with fuscous at incisures. Male unknown.

#### Distribution in Argentina.

Buenos Aires: Delta (58°17'37.0644"S, 58°17'37.0644"W); Chaco: General Vedia (26°56'0.153"S, 58°39'42.015"W), Río Oro (26°56'6.0792"S, 58°40'19.5564"W); Corrientes: Colonia Carlos Pellegrini (28°32'5.4312"S, 57°10'27.5196"W).

### Pnirontis edentula

Taxon classificationAnimaliaHemipteraReduviidae

(Berg)

Centromelus edentulus Berg, 1879: 275; Coscarón 1998: 4.
Pnirontis edentula Lethierry & Severin, 1896: 81; Barber 1930: 171; Wygodzinsky 1949: 68; Maldonado Capriles 1990: 525.
Pnirontes (Centromelus) edentulus Pennington, 1921: 22.

#### Diagnosis.

(After Barber 1930) Scapus shorter than pronotum and about twice as long as preocular margin of head. Antenniferous tubercles long, about 1/4 longer than eye. Pronotum longer than wide.

#### Material examined.

Buenos Aires: 1♀ (MLP) (unspecified locality). Corrientes: 1♀ (MLP) San Roque (28°34'31.1736"S, 58°42'31.032"W). Between Corrientes and Formosa provinces: 1♂ 1♀ (MLP) (unspecified locality). Formosa: 2♂ (MLP) Laguna Oca (26°14'0.0234"S, 58°11'59.9742"W).

#### Distribution in Argentina.

Argentina: Buenos Aires.

#### Observation.

First record for Corrientes and Formosa provinces.

### Pnirontis infirma

Taxon classificationAnimaliaHemipteraReduviidae

Stål

Pnirontis infirma Stål, 1859: 382.

#### Diagnosis.

(After Barber 1930) Scapus shorter than pronotum and about twice as long as preocular margin of head. Pronotum almost as wide as long. Antenniferous tubercles shorter, about equal to length of eyes.

#### Material examined.

Chaco: 1♀ (MLP) Resistencia (27°27'23.3742"S, 58°58'55.776"W); Jujuy: 2♂ 2♀ Reyes (MLP) (unspecified locality); Santa Fe: 1♂ (MLP) Colonia Mascías (30°47'55.8348"S, 60°0'52.3218"W); Santiago del Estero: 1♂ (MLP) Beltrán (27°49'43.6506"S, 64°3'35.5068"W).

#### Observation.

New record for Argentina.

### Pnirontis scorpiona

Taxon classificationAnimaliaHemipteraReduviidae

(Berg)

Centromelus scorpionius Berg, 1879: 276; Coscarón 1998: 6.
Pnirontes (Centromelus) scorpionius Pennington, 1921: 22.
Pnirontis corpionia Barber, 1930: 161; Wygodzinsky 1949: 68; Giacchi 1985: 65; Maldonado Capriles 1990: 526; Coscarón 2003: 361.
Pnirontis scorpionica Lethierry & Severin, 1896: 81.
Pnirontis (Centromelus) scorpioni Giacchi, 1988a: 6.
Pnirontis scorpionia Carpintero & De Biase, 2011: 35

#### Diagnosis.

(After Barber 1930, Giacchi 1985) Female head with tylus produced into a single process. Juga minute. Scapus longer than head. Genae well extended beyond apex of antenniferous tubercles. Anterior trochanters armed with a spine. Foretibiae with two series of spines, an inner series of 7–8 spines and an outer series of 4 spines. Corium and connexivum immaculate.

#### Material examined.

Buenos Aires: 1♀ (MLP) Buenos Aires City. Chaco: 1♀ (MLP) Resistencia. Formosa: 1♀ (MLP) (unspecified locality). Santiago del Estero: 1♀ (MLP) (unspecified locality).

#### Distribution in Argentina.

Buenos Aires: Baradero (33°48'30.4704"S, 59°30'19.6986"W), Buenos Aires City (34°36'13.5102"S, 58°22'53.4678"W), Isla Martín García (34°10'53.6154"S, 58°15'5.6592"W); Chaco: Resistencia (27°27'23.3742"S, 58°58'55.776"W); Córdoba: Sierras (31°26'20.4678"S, 64°50'4.0992"W); Corrientes: Estación Puerto Valle (29°2'0.225"S, 59°11'31.113"W), Ituzaingó (27°40'30.8742"S, 56°48'13.9428"W), San Cayetano (27°34'14.9988"S, 58°41'40.9986"W); Entre Ríos: Victoria (32°37'18.9048"S, 60°9'27.3312"W); Santa Fe: San Cristóbal (30°18'30.2142"S, 61°14'19.9176"W).

### Pnirontis stali

Taxon classificationAnimaliaHemipteraReduviidae

(Mayr)

Pnirontis (Centromelus) stali Mayr, 1865: 437; Melo et al. 2004: 61.
Centromelus stali Berg, 1879: 295.
Pnirontes (Centromelus) stali Pennington, 1921: 22.

#### Diagnosis.

Translated from Mayer (1865): Genae slightly longer than antenniferous tubercles. Scapus spiny underneath and almost 1/3 longer than the head. Pale yellow, in part dark, abdominal margin with small dark spots.

#### Material examined.

1♀ (MLP), 3♂ (MLP) Geographic origin not given.

#### Distribution in Argentina.

Buenos Aires: Chacabuco (34°38'22.4304"S, 60°28'9.9726"W); Corrientes: Colonia Carlos Pellegrini (28°32'5.4312"S, 57°10'27.5196"W); Misiones.

### Pnirontis tabida

Taxon classificationAnimaliaHemipteraReduviidae

Stål

Pnirontis tabida Stål, 1859: 381.
Pnirontis (Centromelus) tabida Melo et al., 2004: 61.

#### Diagnosis.

(After Barber 1930) Female tylus extending into a single stout process beyond apex of antenniferous tubercles. Juga very short, scarcely visible. Scapus, including long apical spine, 1/4 longer than head. Genae short, extending but little beyond apex of antenniferous tubercles. Foretibiae armed only with an inner series of spines and with preapical spur; corium and connexivum immaculate.

#### Distribution in Argentina.

Argentina: Corrientes: Colonia Carlos Pellegrini (28°32'5.4312"S, 57°10'27.5196W).

### Pygolampis

Taxon classificationAnimaliaHemipteraReduviidae

Germar

Pygolampis Germar, 1817: 286.

#### Type species.

*Acanthia denticulata* Rossi, Junior synonym of *Cimex bidentatus* Goeze, 1778.

#### Diagnosis.

(After Barber 1930) Scapus not produced beyond insertion of basiflagellomere. First labial segment approximately twice as long as second and third segments. Scapus unarmed beneath. Head dorsally armed with two prominent tubercles.

### Pygolampis pectoralis

Taxon classificationAnimaliaHemipteraReduviidae

(Say)

Reduvius pectoralis Say, 1832: 11.
Pygolampis pectoralis Pennington, 1921: 22.

#### Diagnosis.

(After Barber 1930) Scapus little if any longer than head. Head just behind eyes armed with a large ramose spine, followed by one or two smaller ones.

#### Distribution in Argentina.

Misiones.

### Pygolampis spurca

Taxon classificationAnimaliaHemipteraReduviidae

Stål

Pygolampis spurca Stål, 1859: 379.

#### Diagnosis.

(After Barber 1930) Scapus twice or as long as head. Basiflagellomere finely pilose with setae longer than diameter of the segment.

#### Material examined.

Catamarca: 1♂ (MLP) Catamarca City (28°28'8.367"S, 65°46'44.2986"W), 1♀ (MLP) (unspecified locality); Corrientes: 1♀ (MLP) Santo Tomé (28°33'0.6696"S, 56°2'56.8062"W), 1♀ (MACN) Manantiales (27°55'28.0704"S, 58°6'9.7914"W); Formosa: 1♀ (MLP) (unspecified locality); Misiones: 1♂ (MACN) (unspecified locality); Santiago del Estero: 1♂ (MLP) Río Salado (unspecified locality).

#### Observation.

New record for Argentina

### Seridentus

Taxon classificationAnimaliaHemipteraReduviidae

Osborn

Seridentus Osborn, 1904: 195.

#### Type species.

*Seridentus denticulatus* Osborn, 1904.

#### Diagnosis.

(After Maldonado Capriles 1994a) Anteocular space as long as or slightly shorter than postocular space. Two lines of setigerous tubercles on ventral side of head slightly surpassing the anterior and posterior margins of eyes. Spines about half as long as posteroventral setigerous tubercles behind eyes. Scutellar spine angulate, raised or vertical. Profemur moderately incrassate. Anterior legs with third tarsal segment twice as long as first and second combined.

### Seridentus latissimus

Taxon classificationAnimaliaHemipteraReduviidae

Giacchi

Seridentus latissimus Giacchi, 1998: 31.

#### Diagnosis.

(After Giacchi 1998) Scapus three times as long as anteocular region of head.

Lateral margins of pronotum with a row of small to setigerous tubercles. Pronotum less.

Pronotum less than twice as long as head. Juga and scutellar spines nearly porrect.

#### Distribution in Argentina.

Misiones: Iguazú (25°57'2.289"S, 54°12'43.329"W).

### Seridentus maculosus

Taxon classificationAnimaliaHemipteraReduviidae

(Haviland)

Seridentus maculosus Haviland, 1931: 136.
Seridentus maculosus : Wygodzinsky, 1949: 69.

#### Diagnosis.

(After Maldonado Capriles 1994a) Scapus twice as long as anteocular region of head. Lateral margins of pronotum with a row of small setigerous tubrecles. Pronotum less than twice as long as head. Juga and scutellar spines nearly porrect. Clavus, corium and membrane sparsely spotted with brown.

#### Material examined.

Misiones: 1♀ (MACN) Iguazú (25°57'2.289"S, 54°12'43.329"W).

#### Observation.

New record for Argentina.

### Stenopoda

Taxon classificationAnimaliaHemipteraReduviidae

Laporte

Stenopoda Laporte, 1832: 26.

#### Type species.

*Stenopoda cinerea* Laporte, 1832.

#### Diagnosis.

(After Barber 1930, Giacchi 1969, Giacchi 1988b) First labial segment shorter than the second and third segments combined. Postocular region shorter than preocular one. Body and appendages with dense, adpressed pubescence and numerous tiny, erect bristles. Margins of head nearly parallel-sided in dorsal view, abruptly constricted at neck. Two median dorsal carinae (1+1) more or less elevated.

### Stenopoda (Megastenopoda)

Taxon classificationAnimaliaHemipteraReduviidae

Giacchi

Stenopoda (Megastenopoda) Giacchi, 1988b: 48.

#### Type species.

### Stenopoda subinermis

Taxon classificationAnimaliaHemipteraReduviidae

Stål, 1859: 384.

#### Diagnosis.

(After Giacchi 1988b) Total length of 23 to 35 mm. Fossula spongiosa of 1/3 to 1/2 the length of the foretibiae.

### Stenopoda cana

Taxon classificationAnimaliaHemipteraReduviidae

Stål

Stenopoda cana Stål, 1859: 384; Giacchi 1969: 11; Maldonado Capriles 1990: 540.

#### Diagnosis.

(After Giacchi 1988b) Abdominal segments 1–5 divergent, the rest convergent. Pronotal setae longer than tubercles height. Tubercles conical and thick.

#### Material examined.

Santiago del Estero: 1♂ (MLP) (unspecified locality).

#### Distribution in Argentina.

Misiones: Puerto Iguazú (25°35'50.895"S, 54°34'42.873"W).

#### Observation.

First record for Santiago del Estero province.

### Stenopoda lativentris

Taxon classificationAnimaliaHemipteraReduviidae

Giacchi

Stenopoda lativentris Giacchi, 1969: 13; Bachmann 1999: 215, 224.

#### Diagnosis.

(After Giacchi 1988b) Abdominal segments 1–5 divergent, the rest convergent. Pronotal setae two times longer than tubercles height. Subcylindrical tubercles, longer than wide.

#### Distribution in Argentina.

Misiones: Pindapoy (27°45'2.592"S, 55°47'28.4856"W), Puerto Iguazú (25°35'50.6862"S, 54°34'43.4922"W).

### Stenopoda pallida

Taxon classificationAnimaliaHemipteraReduviidae

Giacchi

http://research.amnh.org/iz/types_db/images/Stenopoda_pallida.jpg

Stenopoda pallida Giacchi, 1969: 13; Giacchi 1988b: 56.

#### Diagnosis.

(After Giacchi 1988b) Side of the abdomen parallel. Pronotal setae curved and shorter than height of tubercle. Tubercles semispherical. Fossula spongiosa of 1/5 or 1/6 foretibia length.

#### Distribution in Argentina.

Misiones: Eldorado (26°25'1.506"S, 54°36'41.3706"W); Río Negro: El Bolsón (41°58'10.9236"S, 71°32'14.3694"W).

### Stenopoda subinermis

Taxon classificationAnimaliaHemipteraReduviidae

Stål

Stenopoda subinermis Stål, 1859: 384; Melo et al. 2011: 7.

#### Diagnosis.

(After Giacchi 1988b). Sides of the abdomen parallel. Pronotal setae shorter than tubercles height. Semispherical tubercles. Foretibiae with fossula spongiosa of equal length to half the length of the tibia.

#### Material examined.

Chaco: 1♂ (MLP) Chaco National Park.

#### Distribution in Argentina.

Chaco: Chaco National Park (26°48'24.9984"S, 59°26'36.4986"W).

### Stenopoda (Stenopoda)

Taxon classificationAnimaliaHemipteraReduviidae

Giacchi, 1988

Stenopoda (Stenopoda) Giacchi, 1988b: 48.

#### Type species.

*Stenopoda cinerea* Laporte, 1832.

#### Diagnosis.

(After Giacchi 1988b) Total length of 18 to 26 mm. Fossula spongiosa of 1/7 to 1/4 the length of the foretibiae.

### Stenopoda cinerea

Taxon classificationAnimaliaHemipteraReduviidae

Laporte

Stenopoda cinerea Laporte, 1832: 26; Barber 1930: 203; Quintanilla et al. 1976: 129; Froeschner 1988: 648.

#### Diagnosis.

(After Giacchi 1988b) Spots of the connexival segments dark brown, ellipsoidal. Fossula spongiosa of 1/5 to 1/4 the length of the foretibiae.

#### Material examined.

Chaco: 1♀ (MLP) Fontana (27°25'1.0452"S, 59°1'54.6882"W); Santiago del Estero: 1♂ (MLP) (unspecified locality).

#### Distribution in Argentina.

Chaco: Fontana; Córdoba: as south as Córdoba; Corrientes: Departamento Monte Caseros (30°15'9.4212"S, 57°37'20.604"W), Departamento San Luis del Palmar (27°30'40.464"S, 58°33'30.4518"W).

#### Observation.

First record for Chaco and Santiago del Estero provinces.

### Stenopoda guaranitica

Taxon classificationAnimaliaHemipteraReduviidae

Giacchi

Stenopoda guaranitica Giacchi, 1969: 19; Giacchi 1988b: 52; Maldonado Capriles 1990: 541; Bachmann 1999: 214; Coscarón 2003: 361.

#### Diagnosis.

(After Giacchi 1988b) Total length between 18 and 26 mm. Pronotal setae one and a half times longer than tubercles height. Fossula spongiosa of 1/7 to 1/5 the length of the foretibiae.

#### Material examined.

Río Negro: 1♂ (MLP) Pemona (39°29'9.2142"S, 65°36'33.5592"W); Formosa: 2♂ (MLP) Isla Oca (26°15'13.6722"S, 58°11'15.846"W), 1♀ (MLP) Río Paraj. 1♀ (unspecified province and locality), 3♂ (unspecified locality).

#### Distribution in Argentina.

Chaco: Apóstol, Resistencia (27°26'37.0356"S, 58°58'7.8924"W), Río de Oro (58°40'19.5564"S, 58°40'19.5564"W); Córdoba; Corrientes: Garruchos (28°10'23.3076"S, 55°39'18"W), Ituzaingó (27°35'41.532"S, 56°41'56.022"W), Santo Tomé (28°32'51.507"S, 56°2'14.3232"W); Entre Ríos: El Palmar (31°51'51.5808"S, 58°12'30.5346"W); Formosa: El Coatí (25°43'59.8794"S, 59°37'59.8794"W), Palo Santo (25°33'49.7304"S, 59°20'10.5252"W); Jujuy: Calilegua (23°46'28.221"S, 64°46'16.575"W); Mendoza; Misiones: Arroyo Uruguaí (25°53'32.157"S, 54°35'58.1136"W), Eldorado (26°25'1.506"S, 54°36'41.3706"W), Iguazú (25°36'40.0062"S, 54°35'14.067"W), Montecarlo (26°34'21.5646"S, 54°46'1.8042"W), Posadas (27°22'50.1918"S, 55°54'51.8472"W), Zaimán (27°25'6.801"S, 55°53'40.47"W), Departamento Concepción: Panembí (27°43'36.48"S, 54°54'54.5394"W), Pindapoy (27°45'2.592"S, 55°47'28.4856"W); Santa Fe: Departamento De Garay: Colonia Mascías (30°47'55.8348"S, 60°0'52.3218"W); Salta: Departamento Anta: La Forestal (24°55'0.0114"S, 64°28'0.0012"W), Metán (25°29'47.4318"S 64°58'19.3044"W), Salta City (24°47'47.5902"S 65°23'33.666"W), Las Delicias (23°56'1.0428"S, 63°19'51.096"W), Urundel (23°33'28.8288"S, 64°23'50.9994"W), Departamento Orán: Tablillas (22°38'0.0306"S, 63°51'0.1038"W), La Candelaria (26°6'4.554"S, 65°5'59.0814"W); Santiago del Estero: Colonia Mackinlay (30°22'0.9546"S, 62°7'0.8754"W); Tucumán: San Pedro de Colalao (26°14'4.2504"S, 65°29'19.9674"W).

#### Observation.

First record for Río Negro province.

### Stenopoda wygodzinskyi

Taxon classificationAnimaliaHemipteraReduviidae

Giacchi

http://research.amnh.org/iz/types_db/images/Stenopoda_wygodzinskyi.jpg

Stenopoda wygodzinskyi Giacchi, 1969: 19; Maldonado Capriles, 1990: 541; Coscarón 2003: 61.

#### Diagnosis.

(After Giacchi 1988b) Setae of scapus of length equal to its diameter. Pronotal setae three times longer than tubercles height. Fossula spongiosa of 1/7 to 1/6 the length of the foretibiae.

#### Distribution in Argentina.

Corrientes: Santo Tomé (28°33'6.6378"S, 56°2'43.52"W).

## Supplementary Material

XML Treatment for Ctenotrachelus

XML Treatment for Ctenotrachelus minor

XML Treatment for Ctenotrachelus striatus

XML Treatment for Ctenotrachelus testaceus

XML Treatment for Diaditus

XML Treatment for Diaditus latulus

XML Treatment for Diaditus pilosicornis

XML Treatment for Diaditus semicolon

XML Treatment for Gnathobleda

XML Treatment for Gnathobleda fraudulenta

XML Treatment for Gnathobleda litigiosa

XML Treatment for Gnathobleda toba

XML Treatment for Gnathobleda violenta

XML Treatment for Narvesus

XML Treatment for Narvesus carolinensis

XML Treatment for Narvesus minor

XML Treatment for Ocrioessa

XML Treatment for Ocrioessa cornutulus

XML Treatment for Oncocephalus

XML Treatment for Oncocephalus hirsutus

XML Treatment for Oncocephalus maiusculus

XML Treatment for Oncocephalus validispinis

XML Treatment for Pnirontis

XML Treatment for Pnirontis (Centromelus)

XML Treatment for Pnirontis acuminata

XML Treatment for Pnirontis edentula

XML Treatment for Pnirontis infirma

XML Treatment for Pnirontis scorpiona

XML Treatment for Pnirontis stali

XML Treatment for Pnirontis tabida

XML Treatment for Pygolampis

XML Treatment for Pygolampis pectoralis

XML Treatment for Pygolampis spurca

XML Treatment for Seridentus

XML Treatment for Seridentus latissimus

XML Treatment for Seridentus maculosus

XML Treatment for Stenopoda

XML Treatment for Stenopoda (Megastenopoda)

XML Treatment for Stenopoda subinermis

XML Treatment for Stenopoda cana

XML Treatment for Stenopoda lativentris

XML Treatment for Stenopoda pallida

XML Treatment for Stenopoda subinermis

XML Treatment for Stenopoda (Stenopoda)

XML Treatment for Stenopoda cinerea

XML Treatment for Stenopoda guaranitica

XML Treatment for Stenopoda wygodzinskyi
